# Approaches towards DNA Vaccination against a Skin Ciliate Parasite in Fish

**DOI:** 10.1371/journal.pone.0048129

**Published:** 2012-11-07

**Authors:** Louise von Gersdorff Jørgensen, Jens Sigh, Per Walter Kania, Lars Holten-Andersen, Kurt Buchmann, Theodore Clark, Jesper Skou Rasmussen, Katja Einer-Jensen, Niels Lorenzen

**Affiliations:** 1 Department of Veterinary Disease Biology, University of Copenhagen, Frederiksberg, Denmark; 2 Department of Microbiology and Immunology, Cornell University, Ithaca, New York, United States of America; 3 National Veterinary Laboratory, Technical University of Denmark, Aarhus, Denmark; The Scripps Research Institute and Sorrento Therapeutics, Inc., United States of America

## Abstract

Rainbow trout (*Oncorhynchus mykiss*) were immunized with plasmid DNA vaccine constructs encoding selected antigens from the parasite *Ichthyophthirius multifiliis*. Two immobilization antigens (I-ags) and one cysteine protease were tested as genetic vaccine antigen candidates. Antigenicity was evaluated by immunostaining of transfected fish cells using I-ag specific mono- and polyclonal antibodies. *I. multifiliis* specific antibody production, regulation of immune-relevant genes and/or protection in terms of parasite burden or mortality was measured to evaluate the induced immune response in vaccinated fish. Apart from intramuscular injection, needle free injection and gene gun delivery were tested as alternative administration techniques. For the I-ags the complement protein fragment C3d and the termini of the viral haemorrhagic septicaemia virus glyco(G)protein (VHSV G) were tested as opsonisation and cellular localisation mediators, respectively, while the full length viral G protein was tested as molecular adjuvant. Expression of I-ags in transfected fish cells was demonstrated for several constructs and by immunohistochemistry it was possible to detect expression of a secreted form of the Iag52B in the muscle cells of injected fish. Up-regulations of mRNA coding for IgM, MHC I, MHC II and TCR β, respectively, were observed in muscle tissue at the injection site in selected trials. In the spleen up-regulations were found for IFN-γ and IL-10. The highest up-regulations were seen following co-administration of I-ag and cysteine protease plasmid constructs. This correlated with a slight elevation of an *I. multifiliis* specific antibody response. However, in spite of detectable antigen expression and immune reactions, none of the tested vaccination strategies provided significant protection. This might suggest an insufficiency of DNA vaccination alone to trigger protective mechanisms against *I. multifiliis* or that other or additional parasite antigens are required for such a vaccine to be successful.

## Introduction

The globally expanding aquaculture industry is in need of effective vaccines to combat various severe diseases. The vast majority of existing vaccines target bacterial diseases and only chemical or medical treatments are available against parasitic infections. White spot disease caused by the parasite *I. multifiliis* is a major obstacle for the production of fresh water fish [1]. No commercial vaccine is yet available but research for development of effective vaccines against *I. multifiliis* is ongoing. Fish can acquire protective immunity against this parasitosis [2]–[6]. Non-lethal infections and intra-peritoneal injections of live theronts have been shown to confer immunity [6]–[8]. However, due to the impossibility of cultivating the parasite for large-scale production and the infection risks associated with live vaccines, a recombinant vaccine is required for vaccination under commercial aquaculture conditions. Among recombinant vaccines, DNA vaccines have the advantages of being easy to produce and also being capable of inducing both a cellular and a humoral immune response whereas protein based vaccines may only induces an antibody response [7], [9]. So far, only 4 DNA vaccines have been commercialized and all of them are in the field of veterinary medicine [10]. Among these, one is for protection of fish against the viral disease infectious haematopoetic necrosis (IHN) in Atlantic salmon, caused by the rhabdovirus IHNV [11]. The high efficacy of the experimental DNA vaccines against fish rhabdoviruses [12], [13], including the viral haemorrhagic septicaemia virus (VHSV), warrants testing of a similar vaccination strategy against other infections in fish. These DNA vaccines are able to induce a high level of protection following intramuscular injection of naked DNA without adjuvant [14], [15]. The vaccine plasmids encode the viral glyco(G)protein of the respective viruses. When the G protein is expressed *in situ* by the host cells post intramuscular injection of purified plasmid DNA, a nonspecific antiviral immune response is initially generated followed later by a specific immune response [13], [16].

For *I. multifiliis*, it has been discovered that a group of highly abundant surface membrane proteins on the parasite called immobilization antigens (I-ags) play an important role in the induction of immunity [17]–[19]. Cross-binding of these proteins by antibodies causes the parasites to prematurely exit an immune host [8], [20], [21]. This makes the I-ags potential vaccine candidates and some immunization trials have confirmed their potential as efficient inducers of immunity [22]–[24]. However, it has been shown that immunizations with an I-ag without adjuvant did not induce protection [23]. The genes encoding I-ags Iag52A and Iag52B have previously been inserted into DNA vaccine plasmids followed by protein expression analysis in transfected cells. Initial vaccination trials in channel catfish (*Ictalurus punctatus*) induced a specific antibody response [25]. In the present study, DNA vaccination trials were done with plasmids encoding the two I-ags variants as well as an *I. multifiliis* cysteine protease (ICP2), which has a highly up-regulated expression in the feeding and the infective stage of the parasite life cycle [26] and probably plays an important role in the infection process.

Tested DNA vaccine constructs encoded I-ags and ICP2 (membrane bound or secreted), viral haemorrhagic septicaemia virus glyco(G)protein (VHSV G), as well as combinations thereof. For the I-ags the complement protein fragment C3d was tested as opsonization-mediator, while a DNA vaccine encoding the full length viral G protein was tested as molecular adjuvant. Apart from intramuscular injection, needle free injection and gene gun delivery were tested as alternative administration techniques. Gene expression levels, *I. multifiliis* specific antibody production and immunohistochemical (IHC) analyses were investigated for selected experiments. From these vaccination trials gene regulations were observed, an *I. multifiliis*-specific antibody response was detected, *in situ* expression in muscle sections was seen but no protective response was observed.

## Materials and Methods

### Ethics statement

The Committee for Animal Experimentation, Ministry of Justice, Copenhagen, Denmark, approved the study including the fish rearing and experimentation (license number 2006/561–1204), which was performed following the ethical guidelines listed in the license.

In total, 6 vaccination and challenge trials (T1–T6) were performed. Experimental designs with respect to fish size, temperature, fish density, vaccine groups and dose, sample and time points are summarized in Table 1.

**Table 1:** Experimental design.

Trial No	Plasmid injected	Volume of	Sample	Time point of	Challenge
		vaccine dose	type	sampling	
**T1:** Fish 7.6 g,	Grp 1: Untreated	2×10 µl im	Blood,	10 fish sampled	20 fish from each of the
55 fish/160 L	Grp 2: 10 µg pcDNA3.1	2×10 µl im	liver,	before vaccination,	5 groups.
(except Grp 1:	Grp 3: 10 µg pcDNA3.1-CassN-Iag52A	2×10 µl im	spleen,	while 5 fish per	Challenge 49 dpi: 5000
65 fish/group)	Grp 4: 10 µg pcDNA3.1-CassN-ICP2	2×10 µl im	injection	group were sampled	theronts/fish, 6 h.
Temp: 15–	Grp 5: 5 µg pcDNA3.1-CassN-Iag52A	2×10 µl im	site	at day 1, 14, 32, 47,	Count: 7 dpch
17°C	+5 µg pcDNA3.1-CassN-ICP2			50, 57	
**T2:** Fish 47 g,	Grp 1: Untreated	2×50 µl im	Blood	10 fish sampled	15 fish from each of the
55 fish/160 L	Grp 2: 25 µg pcDNA3.1	2×50 µl im		before vaccination,	6 groups.
(except Grp 1:	Grp 3: 25 µg pcDNA3.1-CassN-Iag52B	2×50 µl im		while 5 fish per	Challenge 47 dpi: 7000
75 fish/group)	Grp 4: 25 µg pcDNA3.1-CassN-ICP2	2×50 µl im		group were sampled	theronts/fish, 4 h.
Temp: 15–	Grp 5: 12.5 µg pcDNA3.1-CassN-	2×50 µl im		at day 1, 14, 32, 39,	Count: 6 dpch
17°C	Iag52A+12.5 µg pcDNA3.1-CassN-ICP2			53	
	Grp 6: 12.5 µg pcDNA3.1-CassN-	2×50 µl			
	Iag52A+12.5 µg pcDNA3.1-CassN-ICP2	subepidermally			
**T3:** Fish 5 g,	Grp 1: 10 µg pVax	10 µl im	Blood,	4 fish per group	Remaining fish of the
55 fish/40 L	Grp 2: 10 µg pcDNA3-vhsG	10 µl im	injection	were sampled day	5 groups (8–13 fish
Temp: 11–	Grp 3: 10 µg pVax-CassL-Iag52B-EEF	10 µl im	site	20 and 58 dpi, as	per group) were mixed.
14°C	Grp 4: 10 µg pcDNA3.1-CassN-ICP2	10 µl im		well as 7 dpch ( = 87	Challenge 80 dpi: 7000
	Grp 5: 10 µg pVax-CassL-Iag52B-EEF	10 µl im		dpi). Fin clipped 44	theronts/fish, 4 h.
	+10 µg pcDNA3.1-CassN-ICP2			dpi	Count: 6 dpch
**T4:** Fish 5.9 g,	Grp A: 2.5 µg pcDNA3.1-Iag52A+2.5	2×25 µl im (2.5 µg in each side)			*I. multifiliis* challenge
10 fish/group	µg pcDNA3.0-vhsG				30 dpi. From 22 to 44
Temp: 11–	Grp B: 2.5 µg pcDNA3.1-Iag52A+2.5				dpch all fish had died
12°C	µg pcDNA3.0				and no difference was
	Grp C: 2.5 µg pcDNA3.1-Iag52B+2.5 µg				observed between
	pcDNA3.0-vhsG				groups.
	Grp D: 2.5 µg pcDNA 3.1-Iag52B+2.5				
	µg pcDNA3.0				
	Grp E: 5 µg pcDNA3.0				
**T5:**	Grp A: 75 µg pcDNA3.0-CassL-Iag52B	25 µl im			*I. multifiliis* challenge
Fish 4.7 g, 20	Grp B: 37.5 µg pcDNA3.0-CassL-				45 dpi. All fish died 3–
fish/group	Iag52A+37.5 µg pcDNA3.0-CassL-				10 dpch and no
Temp: 11–	Iag52B				difference was
12°C	Grp C: PBS				observed between
	Grp D: 50 µg pcDNA 3-CassL-Iag52B	Needle free (Panjet)			the groups.
	Grp E: 20 µg pcDNA3.0-CassL-Iag52A+				
	25 µg pcDNA3.0-CassL-Iag52B				
	Grp F: PBS				
**T6:** Fish	Grp A: 100 µg pcDNA3.1-Iag52A+	25 µl im			*I. multifiliis* challenge
weight 6–	pcDNA3.1-Iag52B				45 dpi. All fish died
8 g, 38	Grp B: 100 µg pVax-CassL-Iag52A-EEF+				from 11–29 dpch and
fish/group	pVax-CassL-Iag52B-EEF				there was no difference
(except Grp F	Grp C: 100 µg pVax-CassL-Iag52A -				between the groups.
and G: 36	3C3d+pVax-CassL-Iag52B-3C3d				
fish/group)	Grp D: 100 µg pcDNA3.0				
Temp: 11–	Grp E: 2.6 µg pcDNA3.1-Iag52A+	Intradermal by gene gun			
12°C	pcDNA3.1-Iag52B				
	Grp F: 2.6 µg pVax-CassL-Iag52A-EEF				
	+pVax-CassL-Iag52B-EEF				
	Grp G: 2.6 µg pVax-CassL-Iag52A-3C3d				
	+pVax-CassL-Iag52B-3C3d				
	Grp H: 2.6 µg pcDNA3.0				

Overview of the experimental DNA vaccination and challenge trials (T1–T6). Fish used were obtained from the Danish Centre for Wild Salmon in Randers and the Salmon Hatchery on Bornholm, Denmark. Grp = group, dpch = days post challenge, dpi = days post injection, temp = temperature.

### Fish

All fish were reared under pathogen-free conditions and transferred to aerated tanks with internal biofilters for 14 days before vaccination. They were fed approximately 1% biomass d^−1^ of pelleted dry feed (Biomar, Denmark).

### Plasmid constructs

The sequences encoding *I. multifiliis* proteins from serotype D (the G5 parasite isolate) [25] used in T1, T2 and T3 (Iag52A, AAK01661, aa 21–468; Iag52B, AAK94941, aa 21–460; ICP2, ABH09756, aa 100–310) were optimized for expression in rainbow trout. In short, two codons, UAA and UAG encode glutamine in ciliates, but function as stop-codons in higher eukaryotic systems. Hence, these stop-codons were changed into CAA and CAG codons encoding glutamine in most organisms including rainbow trout. Plasmids including the *I. multifiliis* sequences optimized for expression in rainbow trout were generated by Genart AG, Germany. The VHSV glycoprotein terminals were used as molecular carrier elements. The general composition of the synthetic genes included from the 5′ end a restriction site for *NheI*, a Kozak sequence, the 5′end of the viral haemorrhagic septicaemia virus glycoprotein gene (vhsG) encoding the N-terminal secretion signal part of the VHSV protein (corresponding to aa 1–40 of Genbank acc.no. CAA46926), the *I. multifiliis* sequence of interest, the C-terminal part of the vhsG (corresponding to aa 450–507), two stop codons and finally a restriction site for *XhoI*. The *I. multifiliis* gene sequence inserts were truncated in the sense that the N-terminal secretion signals were excluded for the Iag52 antigens and the cysteine protease. The three nucleotide sequences were cloned into vector pcDNA3.1 (Invitrogen) using the two above mentioned restriction sites, *NheI* and *XhoI*. Thus, these three plasmid constructs included *I. multifiliis* antigens in a vhsG based cassette for display of the antigens on the cell surface. The plasmids encoding *I. multifiliis* in these vhsG cassettes were designated pcDNA3.1-CassN-IagA (or B) and pcDNA3.1-CassN-ICP2. The CassN term indicates that no linear VHSV G epitope is present in the C-terminal VHSV G domain.

Plasmid constructs used for T3, T4, T5 and T6 included earlier described codon optimised clonings of IagA and IagB in pcDNA3.1 (pcDNA3.1-Iag52A, pcDNA3.1-Iag52B) [25], as well as the derivatives described below. A long vhsG display cassette was established by inserting NheI and ClaI sites at codon positions 39 and 366 respectively in the vhsG sequence using PCR-based mutagenesis. This cassette construct (pcDNA3.0-CassL) has previously been shown to direct cell surface expression of chimaeric fusion proteins (Einer-Jensen, unpublished results), including a linear epitope tag at aa 399–413 recognized by the VHSV G specific MAb IP1H3 [27]. The pcDNA3.0-CassL vector was used to generate constructs encoding either cell surface displayed or secreted variants of the *I. multifiliis* antigens as outlined below. Primers used for PCR cloning are given in Table 2.

**Table 2:** Primers used for PCR-cloning of I-ags into CassL vectors.

Name	Sequence
IAG52A-XbaI	5′>TTTTCTAGAGCTAACTGTCCTGTGGGA
IAG52A-ClaI	5′>TTTATCGATCTGGATGTTCTTCTTAGC
IAG52B-XbaI	5′>TTTTCTAGAGTGAACTGTCCTAACGGA
IAG52B-ClaI	5′>TTTATCGATCTGAGCCTTCTGGGTAGC
EEF-linker(s)	5′>GATCCGAGGAATTCTAAT
EEF-linker(a)	5′>CTAGATTAGAATTCCTCG
vhsG-HindIII	5′>TATAAGCTTACCATGGAATGGAACACT
vhsG-BamHI	5′>TATGGATCCGATTAGGTTGGTGTGGAT
C3d-BglII	5′>TATAGATCTCAGCCAGTCGGGTCTGGG
C3d-BamHI	5′>TATGGATCCGCCGGCCACCTCTAGGTT
C3d-OL(s)	5′>GGGGGATACGGATCAACACAG
C3d-OL(a)	5′>CTGGTGTGATCCGTATCCCCC
G4S-linker(s)	5′>AGCTTAGATCTGGCGGAGGAGGCAGCGGAGGCGGCGGAAGCG
G4S-linker(a)	5′>GATCCGCTTCCGCCGCCTCCGCTGCCTCCTCCGCCAGATCTA

#### pcDNA3.0-CassL-Iag52A and pcDNA3.0-CassL-Iag52B

The ectodomains of *I. multifiliis* I-ags, corresponding to aa 21–449 of Iag52A (GenBank AAK01661) and aa 21–438 of Iag52B (GenBank AAK94941) were amplified by PCR using oligonucleotides Iag52A-XbaI and Iag52A-ClaI for Iag52A and Iag52B-XbaI and Iag52B-ClaI for Iag52B creating XbaI and ClaI restriction sites at the ends. Synthetic immobilization antigen genes [25] were used as template. For the display of the immobilization antigens on the cell surface the Iag52A and Iag52B fragments were inserted into pcDNA3.0-CassL between restriction sites NheI and ClaI replacing aa 40–365 of VHSV G (GenBank AAB26115) creating vaccine constructs pcDNA3.0-CassL-Iag52A and pcDNA3.0-CassL-Iag52B.

#### pVax-CassL-Iag52A-EEF and pVax-CassL-Iag52B-EEF

Secreted forms of the VHSV G-Iag52A and VHSV G-Iag52B fusion proteins were constructed by introducing a stop codon just outside the transmembrane region of VHSV G corresponding to aa 453. First a synthetic oligonucleotide linker of the structure BamHI-EcoRI-Stop-XbaI was made by annealing EEF-linker(s) and EEF-linker(a). This linker was inserted into pVax (Invitrogen, Denmark) between restriction sites BamHI and XbaI to produce vector pVax-EEF. Next fragments encoding the VHSV G-Iag52A or VHSV G-Iag52B fusion proteins were amplified by PCR using oligonucleotides vhsG-HindIII and vhsG-BamHI and pcDNA3.0-CassL-Iag52A or pcDNA3.0-CassL-Iag52B as template. These fragments were inserted into pVax-EEF between restriction sites HindIII and BamHI to produce vaccine constructs pVax-CassL-Iag52A-EEF and pVax-CassL-Iag52B-EEF respectively.

#### pVax-CassL-Iag52A-3C3d and pVax-CassL-Iag52B-3C3d

To improve the immunogenicity of secreted VHSV G-Iag52A and VHSV G-Iag52B fusion proteins, gene constructs encoding three consecutive C3d domains in frame with the 3′ termini of the antigen genes were generated [28]. A DNA fragment coding for rainbow trout complement C3d, corresponding to aa 964–1256 of C3-1 (GenBank AAB05029) was produced by PCR using the cloned C3-1 region as template [29]. By PCR overlap-extension BglII and BamHI sites were added at the ends, Cys-968 was substituted by serine using oligonucleotides C3d-BglII and C3d-BamHI and the internal BamHI site was removed using oligonucleotides C3d-OL(s) and C3d-OL(a). Three copies of this C3d fragment were assembled using a synthetic oligonucleotide linker made by annealing of oligonucleotides G4S-linker(s) and G4S-linker(a) to produce the structure (GS-C3d-GSGGGGSGGGGSGS-C3d-GSGGGGSGGGGSGS-C3d-GS) [28]. The 3C3d fragment was inserted into pVax-CassL-Iag52A-EEF and pVax-CassL-Iag52B-EEF at the BamHI restriction site in the proper orientation to produce pVax-CassL-Iag52A-3C3d and pVax-CassL-Iag52B-3C3d, respectively.

**Figure 1:**
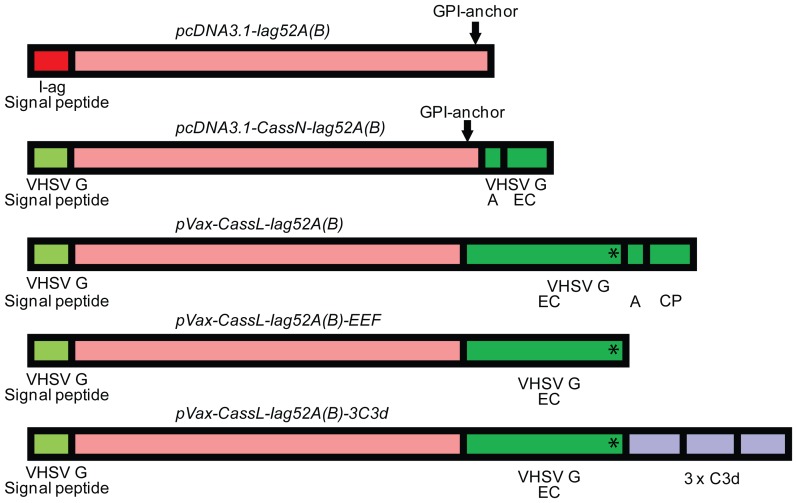
Antigen gene constructs. Schematic presentation of 5 different antigen gene constructs inserted into plasmids used in trial 1–6 including plasmids (pcDNA3.1-Iag52A/B) provided by Drs. Theodore G. Clark and Harry W. Dickerson [25]. The constructs were used in DNA vaccination trials against *I. multifiliis* in rainbow trout. VHSV G = viral haemorrhagic septicaemia virus glyco(G)protein. * Linear VHSV G epitope. EC = extra cellular, A = anchor, CP = cytoplasmic. For further details, see text.

Schematic presentations of the antigen gene constructs are shown in Figure 1. The plasmid construct pcDNA3.0-vhsG has been reported previously [30]. The plasmid backbones pcDNA3.0 as well as pVax refer to either of the commercially available plasmids from Invitrogen.

Batches of plasmids from *E. coli* cultures were affinity purified (Qiagen EndoFree plasmid kit) and the quality was evaluated by electrophoresis using a 1% agarose gel. Finally, the target sequences were sequenced to confirm that no mutations had taken place.

### Transfection assay

To evaluate protein expression from the vaccine plasmids an epithelial cell line derived from an epithelial papilloma in a cyprinid fish (EPC) [31], [32] was transfected according to Heppell et al. (1998) [30]. In brief, 4 µg plasmid in 130 µl Tris-buffered cell culture medium (without serum and antibiotics) and 5,2 µl Superfect (Qiagen) was left for 10 min at room temperature. Then 865 µl cell culture medium containing 10% foetal calf serum (FCS) and antibiotics was added and applied on cells grown in 24 well tissue culture plates. After incubation at 37°C for 2 h, the cells were kept overnight at 28°C. Next morning cells were washed with PBS and incubated in cell culture medium for additional 4 days at 18°C. The transfected EPC cells were fixed using ice cold 80% acetone for 10 min. The cells were then immunostained with rabbit anti-whole cell *I. multifiliis* [33] (1∶500), rabbit anti-G5II Iag52B (1∶500) [34] or mouse MAb G3–61 [34] as primary layer. The rabbit antibodies were subsequently detected by HRP labelled swine anti-rabbit (P217, Dako, Denmark, 1∶300) and mouse MAb G3–61 was detected by HRP conjugated rabbit anti-mouse (P260, Dako, Denmark, 1∶1000). Controls consisted of cells transfected with a plasmid expressing the VHSV G protein (pcDNA3-vhsG [30]). The setups are summarized in Table 3. For examination of antigen location on the outer cell surface, acetone fixation was omitted. Immunostaining followed the same procedure, but at 4°C and with cell culture medium with 15 mM NaN_3_ as incubation and washing buffer. Visualization was done by immunofluorescence. For analyses of temperature effects on the antigenicity of the expressed I-ags, the HEK 293T human embryonic kidney cell line was transfected with selected plasmid constructs and incubated at 24°C or 37°C for 3 days before fixation. The transfection protocol was a modified version of that used for EPC cells in terms of 9 µg plasmid DNA and 20 µl being added into 130 µl Tris-buffered cell culture medium.

**Table 3:** *In vitro* expression studies.

Plasmid construct	Trial	Specific antibodies		
		PAb RαIag	MAb	MAb
			G3–61	IP1H3
pcDNA3.1-Iag52A	T4, T6	−	Nd	Nd
pcDNA3.1-Iag52B	T4, T6	+	+	−
pcDNA3.0-CassL-Iag52A	T5	−	Nd	+
pcDNA3.0-CassL-Iag52B	T5	+	Nd	+
pVax-CassL-Iag52A-EFF	T6	Nd	Nd	+
pVax-CassL-Iag52B-EFF	T3, T6	+	+	+
pVax- CassL-Iag52A-3C3d	T6	−	−	+
pVax- CassL -Iag52B-3C3d	T6	+	+	+
pcDNA3.1-CassN-IAG52A	T1	−	−	−
pcDNA3.1-CassN-IAG52B	T2	−	−	−
pcDNA3.1-CassN-ICP2	T1–3	−	−	−
pcDNA3.0-vhsG	T4	−	−	+
pcDNA3.1-vhsG	T3	−	−	+

The plasmid constructs were transfected into EPC cells and the encoded proteins were subsequently detected using specific primary antibodies: Polyclonal antibody rabbit anti-G5II Iag52B (PAb RαIag), monoclonal mouse *I. multifiliis* (I-ag) specific antibody MAb G3–61, monoclonal antibody VHSV G specific antibody IP1H3 (MAb IP1H3), respectively. The pcDNA3.1-Iag52A (or B) plasmids encoded I-ags encoding their own signal peptides as well as a glycosylphosphatidylinositol (GPI) glycolipid anchor, herby enabling the proteins to attach to the membrane. In the cassette constructs pcDNA3.0-CassL-Iag52A (or B) and pcDNA3.1-CassN-IAG52A (or B) the core I-ag region is flanked by the signal peptide and trimerization- and transmembrane region from the VHSV G protein, respectively. However, the two cassettes differ as CassL includes a linear VHSV G epitope for the MAb IP1H3, whereas this region has been omitted in the CassN construction. The derived pVax-CassL-Iag52A (or B)-3C3d represents secreted I-ag fusion variants including three copies of C3d.

### Vaccination

Fish were anesthetized by immersion in a solution of 80 mg/L of MS222 (Sigma-Aldrich, Denmark) before injection. Intramuscular (im) injections were administered in the epaxial muscle ventrally of the dorsal fin using a 26 gauge needle. Needle free injection was conducted using a Panjet injector (Wright-Cottrell, Scotland [35]), which injects small volumes of liquid using air pressure. Spring strength and spacer was adjusted to levels not inducing macroscopical skin damage. The gene gun is another needle free injection system, where DNA coated gold particles are injected using air pressure. A Helios gene gun (BioRad) was used as outlined elsewhere [36]. Table 1 summarizes the vaccination trials.

### Sampling

Sampling time points are described in Table 1. Blood (T1, T2 and T3): all blood samples were taken from the caudal vein immediately after terminating the fish with an overdose of MS222 using heparinized capillary tubes or heparinized syringes and kept on ice. Plasma was separated from the blood by centrifugation (4000× g for 10 min) and kept at −80°C.

Tissue for qPCR (T1): liver, spleen and muscular tissue from the injection site were collected and immediately placed in RNAlater on ice. These samples were kept at 4°C for 24 hours and then stored at −20°C until RNA purification.

Tissue for immunohistochemistry (T3): all tissues were fixed in cold 4% neutral buffered formaldehyde for 24 hours and then transferred to 70% ethanol until further processing.

### Challenge trials (Further details see Table 1)

Challenge trials 1–3 were conducted with *I. multifiliis* theronts of serotype D. This was confirmed by PCR identification of Iag52B (forward primer: 5′CACAAAGCCGATTCTCAACA, reverse primer: 5′CCAGTAGGACATTCATTGTTAC).

#### T1

The individual weight of the fish was measured and only fish between 5 and 10 g were considered for the analysis. The sum of parasites on the fins 7 days after challenge was divided with the average weight within each group (n = 47, average 7,3 g, range 5–9,7 g). These numbers were used for the comparing analyses between groups. Mortality was registered on a daily basis.

#### T2

The sum of parasites on the fins 6 days after challenge was divided with the length of each fish (n = 36, average 18,6 cm, range 12–22,5 cm). These numbers were used for the comparing analyses between groups. Mortality was registered on a daily basis.

#### T3

After challenge, the fish were randomly placed in 4 aerated 40 L aquaria with biofilters. Seven days later the number of parasites was counted on one side of the fish. The number of parasites were doubled and divided by the length of the fish to account for size differences (n = 56, average 10,7 cm, range 8–13,5 cm).

Challenge trials 4–6 were also conducted with a serotype D isolate of *I. multifiliis* based on sequence homology of Iag52A (forward primer: 5′ATGAAAAATAATATTTTAGTAATATTG, reverse primer: 5′ATTCATCATAATAAATAATAAGAAATC) with that of the G5 isolate. However, detection of Iag52B using another specific PCR primer set (forward primer: ATGAAATTTAATATTTTAATAATTTTG, reverse primer: 5′TCACAACAAATAGAAAGAAATAAATAT) failed for the isolate used in T4–6.

#### T4–6

For these three experiments, the vaccinated fish were challenged by moving the fish to aquaria with an ongoing infection. All aquaria used for challenge contained infected fish with visible mature trophonts and mortality had occurred in the days prior to the challenge. The fish used for maintaining the life cycle of the parasite were removed before transfer of the vaccinated fish. Mortalities were recorded daily during the challenge period.

### Evaluation of I. multifiliis specific antibodies by ELISA

The trout *I. multifiliis* specific antibodies from the three trials (T1–3) were determined using an indirect ELISA with sonicated parasites as coating antigen [37]. In brief, for the production of antigen heavily infected fish were killed by an overdose of MS222 and transferred to a container with tap water. Tomonts were collected with a pipette and transferred to PBS, centrifuged at 4°C for 10 min at 5000× g and washed three times in PBS. Subsequently they were sonicated (5 pulses of: 5 sec sonication, 5 sec pause, 285 W, Heat Systems XL2020, New York, USA). Protein measurement was conducted using a bicinchoninic acid method (Pierce Chemical Co., Illinois, USA). Microtiter plates were coated with 100 µl antigen (sonicated tomonts) in coating buffer (C3041, Sigma-Aldrich, Denmark) overnight at 4°C at a concentration of 5 µg/mL. Free binding sites were blocked with 1% skimmed milk powder (Sigma-Aldrich, Denmark) and 0.2% Tween 20 (Sigma-aldrich, Denmark) in PBS (pH 7.2). Plasma samples from T1 and T2 were diluted 1∶25 whereas samples from T3 were diluted 1∶50 due to plasma limitations. All samples were analyzed in duplicate. Mouse anti-salmonid Ig (MCA2182, AbD Serotec, Germany) was used as secondary antibody (1∶400) and HRP conjugated rabbit anti-mouse IgG (STAR13B, AbD Serotec, Germany) used as tertiary antibody (1∶400). Following incubation with the visualizing reagent tetrametylbenzidine (TMB PLUS) substrate (BUF042B, AbD Serotec, Germany) the reaction was stopped with 1 M HCL. Absorbance was measured at 450 nm. Blood samples collected before and after challenge were analyzed for the detection of antibody levels specific for *I. multifiliis*. For positive controls plasma from immune rainbow trout were used. The immune rainbow trout were produced by subjecting the fish to a continuous natural *I. multifiliis* infection. Plasma samples from T2 were run three times. Specificity of the reactions was examined by running the ELISA without *I. multifiliis* antigen for a representative number of samples.

### Immunohistochemistry (IHC)

Two immunohistochemical studies were conducted for detecting expressed antigen in transfected muscle cells at the injection site. The first study concerned expression of pcDNA3.1-CassN-Iag52A, pcDNA3.1-CassN-Iag52B and pcDNA3.1-CassN-ICP2 and the second study concerned expression of pVax-CassL-Iag52B-EEF and pcDNA3.1-CassN-ICP2. In the first study 8 fish were injected with approximately 25 µg of the different plasmids alone or in combination. Between 6–20 days after injection fish were sampled and the injection site was preserved for IHC analyses. In the second study samples from T3 were used.

Using a standard protocol the muscle tissues were dehydrated in increasing grades of ethanol, cleared in xylene and embedded in paraffin. Then paraffin blocks were sectioned (4 µm) on a Leica RM2135 (Germany) microtome and sections mounted on slides which subsequently were dried at 40°C for 12–24 hours.

After deparaffinization and rehydration different antigen retrieval techniques were used as described in the following section. The antigens were retrieved by exposing the slides to 15 min of microwave oven boiling either in Tris-EDTA buffer (10 mM Tris base, 1 mM EDTA) at pH 9.0 or in citrate buffer (10 mM Citric acid, 0.05% Tween 20) at pH 6.0. Subsequently, the samples were cooled down for a period of 15 min and then subjected to 5 min of tap water washing and transferred to TBS at room temperature. Antigen retrieval was also conducted using proteinase K (0.48 U/ml) treatment at room temperature for 10 min and then 5 min incubation in ice-cold TBS and a following incubation for 5 min in TBS at room temperature.

The antigen specific staining was conducted as described in [33]. Briefly, primary antibodies used in the first study included rabbit anti-whole cell *I. multifiliis* [33], and rabbit anti-papain (SMS gruppen, Denmark) (the cysteine protease ICP2 is papain-like). Primary antibodies used in the second study comprised MAb IP1H3 to VHSV G [38], rabbit anti-whole cell *I. multifiliis* and rabbit anti-G5II Iag52B [34]. Following incubation, unbound primary antibody was gently washed off using TBS and the tissue was covered with either anti-rabbit or anti-mouse EnVision™+System HRP labelled secondary antibody (Dako, Denmark) and left for 30 min incubation at room temperature.

Mayer's haematoxylin (Dako, Denmark) was used as nuclear counter stain and mounting was conducted with aquamount (Merck, UK). The slides were examined using a Leica DMLB microscope (Leica Microsystems, Germany) and images were captured with Leica CD300 (Leica Microsystems, Germany).

The results were evaluated with respect to the finding of cells specifically stained by the immunohistochemical procedures.

### Detection of vaccine plasmids and immune-relevant genes by PCR techniques

#### PCR detection of vaccine constructs

To detect pcDNA3.1-CassN-Iag52A at the injection site in T1 a vector specific primer targeting region T7 (5′TAATACGACTCACTATAGGG3′) was used together with a primer targeting the inserted sequence encoding Iag52A (5′GCTGGGCGCCAGCGTC3′). The expected product size was 332 bp. To detect pcDNA3.1-CassN-ICP2 vector primer T7 was used together with a primer within the inserted sequence encoding ICP2 (5′CGCCGTCACAGCCGTC3′). The expected product size was 797 bp. DNA was purified from tissue at the injection site at all sampling points (DNeasy® Tissue Kit (50), Qiagen, Denmark) and PCR cycling conditions were as follows: initial denaturation at 95°C for 5 minutes; 40 cycles of 95°C for 30 seconds, 55°C for 30 seconds, 72°C for 1 minute; final extension at 72°C for 7 minutes. A reaction volume of 10 µl was used, which contained: 1 µL template (purified DNA); 1 µM of both the forward and the reverse primer; 1 µL of 10× PCR buffer (Bioline, Aarhus, Denmark); 200 µM of dNTP mix (AB, Warrington, UK); 1 unit Taq-polymerase (Bioline, Aarhus, Denmark); 1.5 mM MgCl_2_ (Bioline, Aarhus, Denmark).

#### Gene expression by real-time qPCR

To detect expression of genes encoding parasite antigens and host immune genes at the injection site a series of qPCR analyses were performed. RNA purification, cDNA synthesis and real-time qPCR were conducted according to [6]. The Stratagene MX3000PTM real-time PCR system was used and primers and probes are shown in Table 4. Negative controls included both a mock reverse transcription reaction (RT minus) and a mastermix setup with water instead of template. Elongation factor (EF 1-α) was used as housekeeping gene [39]–[41]. For the expression data regarding the plasmids the value 37 was inserted instead of no CT values when they were encountered (37 was the highest CT value that was obtained).

**Table 4:** Primers and probes used for qPCR.

Gene	Acc. no.	Product	Primer	Probe
		size (bp)		
**Housekeeping gene**				
EF1-α	AF498320	63	Fwd: ACCCTCCTCTTGGTCGTTTC	GCTGTGCGTGACATGA
			Rev: TGATGACACCAACAGCAACA	GGCA
**Plasmid inserts**				
pcDNA3.1-	AAK01661	80	Fwd: CGCCGCTAACTTTTACACCA	AAGCAGACCGACTGG
CassN-Iag52A			Rev: GCTGGTACAGGTGTCGATGC	GTCGC
pcDNA3.1-	ABH09756	93	Fwd: GCTGTCAGGACACCTCCAAG	AGGTCAACATCACCAA
CassN-ICP2			Rev: CCAGAGCCTTCTCAAAGCTG	GTTCGCC
**Complement factors**				
C3	AF271080	85	Fwd: ATTGGCCTGTCCAAAACACA	TGGAATCTGTGTGTCT
			Rev: GCTTCAGATCAAGGAAGAAGTTC	GAACCCC
**Immunoglobulins**				
IgM	S63348	72	Fwd: CTTGGCTTGTTGACGATGAG	TGGAGAGAACGAGCA
			Rev: GGCTAGTGGTGTTGAATTGG	GTTCAGCA
IgT	AY870265	72	Fwd: AGCACCAGGGTGAAACCA	AGCAAGACGACCTCCA
			Rev: GCGGTGGGTTCAGAGTCA	AAACAGAAC
**Cytokines**				
IL-1β	AJ223954	91	Fwd: ACATTGCCAACCTCATCATCG	CATGGAGAGGTTAAAG
			Rev: TTGAGCAGGTCCTTGTCCTTG	GGTGGC
IL-10	AB118099	70	Fwd: CGACTTTAAATCTCCCATCGAC	CATCGGAAACATCTTC
			Rev: GCATTGGACGATCTCTTTCTTC	CACGAGCT
IFN-γ	AY795563	68	Fwd: AAGGGCTGTGATGTGTTTCTG	TTGATGGGCTGGATGA
			Rev: TGTACTGAGCGGCATTACTCC	CTTTAGGA
**Cell receptors**				
TcR beta chain	AF329700	73	Fwd: TCACCAGCAGACTGAGAGTCC	CCAATGAATGGCACAA
			Rev: AAGCTGACAATGCAGGTGAATC	ACCAGAGAA
CD8	AF178054	74	Fwd: ACACCAATGACCACAACCATAGAG	ACCAGCTCTACAACTG
			Rev: GGGTCCACCTTTCCCACTTT	CCAAGTCGTGC
MHC I	AY523661	73	Fwd: TCCCTCCCTCAGTGTCT	CAGAAGACCCCCTCCT
			Rev: GGGTAGAAACCTGTAGCGTG	CTCCAGT
MHC II	AF115533	67	Fwd: TGCCATGCTGATGTGCAG	CGCCTATGACTTCTAC
			Rev: GTCCCTCAGCCAGGTCACT	CCCAAACAAAT
**Acute Phase Proteins**				
Hepcidin	AF281354	95	Fwd: GAGGAGGTTGGAAGCATTGA	AGTCCAGTTGGGGAAC
			Rev: TGACGCTTGAACCTGAAATG	ATCAACAG
SAA	AM422446	93	Fwd: GGGAGATGATTCAGGGTTCCA	TCGAGGACACGAGGA
			Rev: TTACGTCCCCAGTGGTTAGC	CTCAGCA

Primers and probes to detect expression of genes encoding parasite antigens and host immune genes at the injection site (Fwd: forward, Rev: reverse) and their GenBank accession no., product size and sequence. EF: elongation factor, Ig: immunoglobulin, IL: interleukin, IFN: interferon, TcR: T cell receptor, CD: cluster of differentiation, MHC: major histocompatibility complex, SAA: serum amyloid A.

### Statistical analyses

#### T1–T3

Counts of the parasite burden as well as the obtained ELISA results were tested with a one way ANOVA test and a Newman-Keuls post test in Graphpad Prism 5. The gene expression analyses (T1) were determined according to the 2^−ΔΔCt^ method [42]. Expression data are presented as the fold increase or decrease of the vaccinated fish compared to control fish. Fold changes below 3 are not considered [43]. ** indicates significance using a two tailed t-test (p<0.05) and * indicates significance using a one-tailed t-test (p<0.05). Predictions *in silico* of the transmembrane antigens encoded by the plasmids were conducted using Center for Biological Sequence analyses (CBS), Technical University of Denmark (DTU) [44].

## Results

### Verification of antigen expression in transfected fish cells

**Figure 2:**
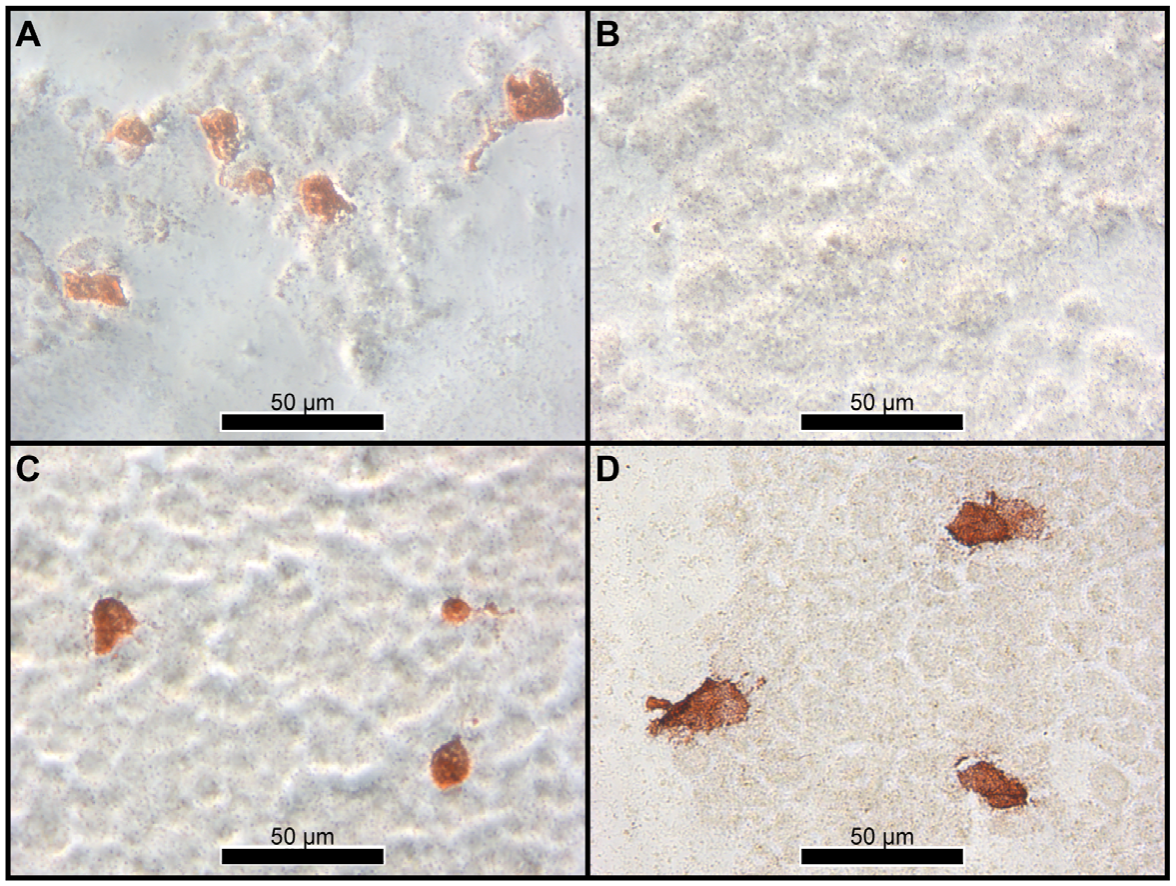
Immunochemical visualization of I-ags expressed in transfected 293 T cells. Cell cultures were transfected with pcDNA3.1-Iag52B (A, B) or with pcDNA3.0-CassL-Iag52B (C, D). Immunostaining was performed using the anti-*I. multifiliis* (I-ag) monoclonal antibody G3–61 (A, C) or the anti-VHSV G monoclonal antibody IP1H3 (B, D) as primary antibodies.

None of the CassN gene constructs used in trials T1–T3 could be verified in terms of protein expression (Table 3). In terms of the ICP2, no specific antibodies are available and the rabbit anti-papain antibody may not cross react. For CassL used in T4–T6, the Iag52B antigen and derivatives thereof could be detected in transfected EPC cells with both MAb G3–61 and rabbit antiserum to Iag52B. In contrast to this, expression of Iag52A antigen and its derivatives could not be detected with the available I-ag specific antibodies. However, all pVax CassL constructs were functionally confirmed in terms of protein expression in transfected cells by staining with MAb IP1H3 recognizing the C-terminal VHSV G domain (Table 3, Fig. 2). Immunostaining of transfected 293 T cells incubated at higher temperatures (24°C and 37°C, respectively) following transfection and fixation in 80% acetone gave similar results as obtained with EPC cells incubated at 18°C (Fig. 2). Since the anti-Iag52 MAb G3–61 recognizes a disulphide dependent conformational epitope, this suggests that the I-ags are correctly folded throughout the tested temperature range.

### Response to vaccination and challenge

**Figure 3:**
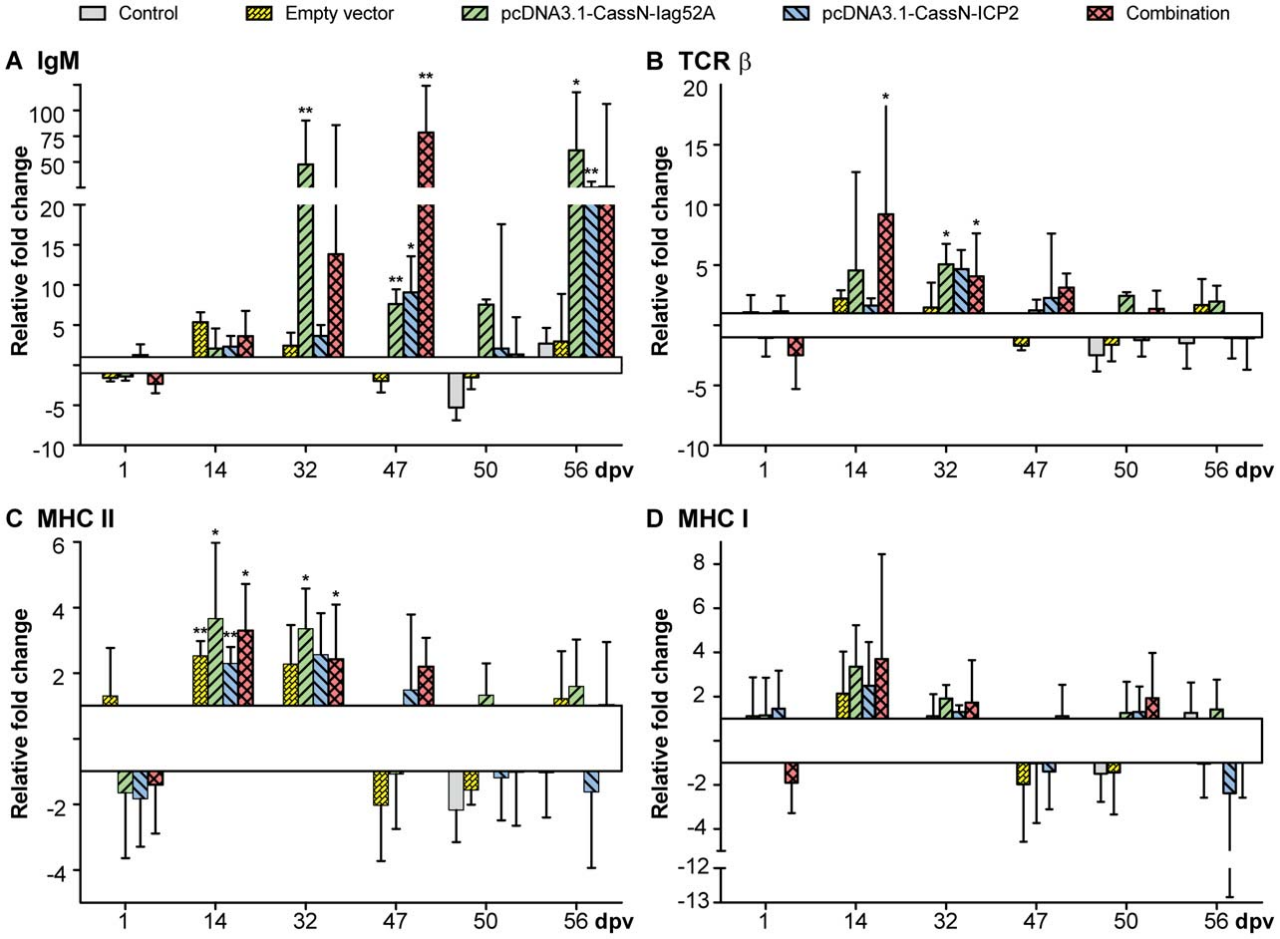
Real time quantitative expression data in muscle tissue from the injection site (T1). Graphs showing real time quantitative expression data of immune relevant genes in muscle tissue sampled at the injection site from T1 in *O. mykiss* following DNA vaccination against *I. multifiliis* with pcDNA3.1-CassN-Iag52A and pcDNA3.1-CassN-ICP2; A) IgM, B) TCR β, C) MHC II and D) MHC I. Challenge was conducted 49 dpv. ** significant with a two-tailed t-test (p<0.05). * significant with a one-tailed t-test (p<0.05).

Results obtained from trial one (T1), where rainbow trout were immunized with plasmids encoding membrane bound Iag52A and ICP2 and subsequently challenged with *I. multifiliis* showed no protective effect against *I. multifiliis*, nor did the ELISA data show any difference in specific antibody levels between the groups. The IHC studies did not show any positively transfected muscle cells at the injection site. However, by PCR it was possible to detect the presence of the plasmids in the muscle tissue throughout the whole trial. Additionally, it was possible to confirm high transcription of the inserted sequences encoding Iag52A and ICP2 by qPCR at the injection site. At the injection site expression of the complement factor C3 and the acute phase protein SAA were not regulated (data not shown). However, expression of IgM, TCR β, MHC I and II were regulated following DNA injection. The expression of IgM was up-regulated 32 dpi for the group injected with pcDNA3.1-CassN-Iag52A (43 fold) and for the combination group (14 fold). At day 47 expression of IgM in all vaccination groups was significantly up-regulated with the combination group reaching 79 fold up-regulation. At day 50 no regulation was observed. However, 56 dpi (7 days after challenge), up-regulation was seen within all vaccination groups (Fig. 3A). Expression of TCR β mRNA (Fig. 3B) increased significantly on day 14 within the group injected with both constructs (9 fold) and 32 dpv an up-regulation was observed for the vaccination groups (5, 5 and 4 (not significant) fold, respectively). A slight up-regulation of MHC I and II was seen for all groups (Fig. 3C, 3D) except the control group on day 14 and for MHC II alone 32 dpv. Regulations of MHC I were non-significant.

**Figure 4:**
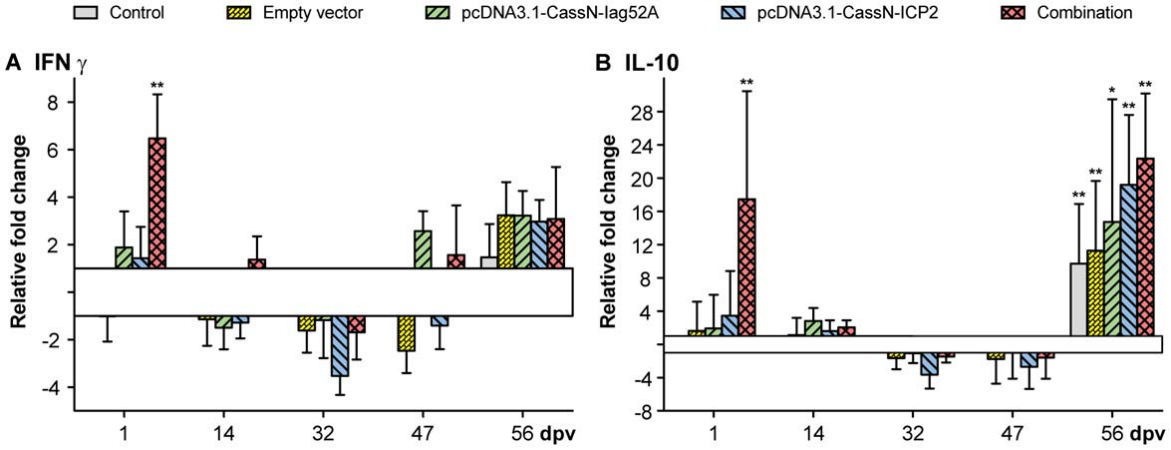
Real time quantitative expression data from spleen collected from T1. The graphs show real time quantitative expression data of immune relevant genes in the spleen of *O. mykiss* following DNA vaccination against *I. multifiliis* with pcDNA3.1-CassN-Iag52A and pcDNA3.1-CassN-ICP2; A) IFN-γ and B) IL-10. Challenge was conducted 49 dpv. ** significant with a two-tailed t-test (p<0.05). * significant with a one-tailed t-test (p<0.05).

In the spleen gene regulation were not seen for C3, IgM, TCR β, MHC II and IL-1 β before challenge. Only the expression of IL-1β was found significantly up-regulated (data not shown) after challenge for all groups with the combination group reaching the highest fold change (18 fold). Gene expression of Iag52A and ICP2 was not detected. The expression of IL-10 and IFN-γ was found to be regulated at day one after vaccination and 7 days post challenge (Fig. 4). An up-regulation within the combination group at day one (17 fold for IL-10 and 6 fold for IFN-γ) was observed and an up-regulation was seen for all groups 56 dpv, 7 days after challenge for IL-10. It is noteworthy that the highest fold increase with regard to IL-10 and IFN-γ one dpv was observed within the combination group.

**Figure 5:**
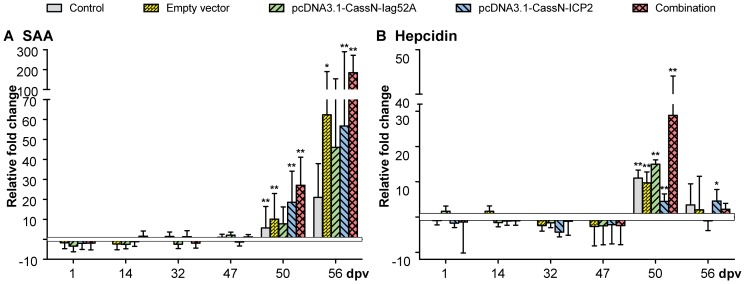
Real time quantitative expression data from liver collected from T1. The graphs show real time quantitative expression data of immune relevant genes in the liver following DNA vaccination of *O. mykiss* against *I. multifiliis* with pcDNA3.1-CassN-Iag52A and pcDNA3.1- CassN-ICP2; A) SAA and B) Hepcidin. Challenge was conducted 49 dpv. ** significant with a two-tailed t-test (p<0.05). * significant with a one-tailed t-test (p<0.05).

In the liver up-regulations of SAA and hepcidin mRNA were only observed after challenge for all groups (Fig. 5). The highest up-regulations were found for the combination group. C3 was not regulated and Iag52A and ICP2 were not expressed.

No protective effect of the vaccines was observed. On the contrary, the group vaccinated with pcDNA3.1-CassN-ICP2 was found to be infected with a significantly higher number of parasites.

**Figure 6:**
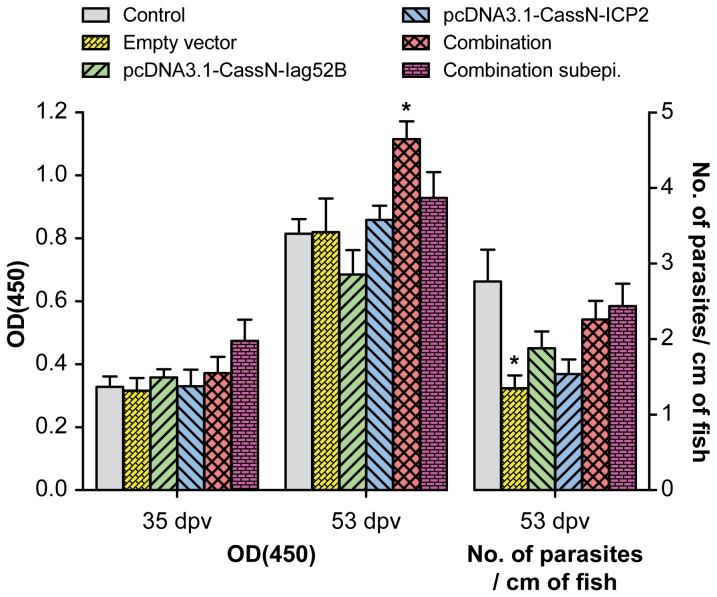
Antibody measurements and parasite load after challenge from T2. The graph shows both the measurement by OD(450) of *I. multifiliis* specific antibodies and the parasite burden of *O. mykiss* following challenge with *I. multifiliis* after DNA vaccination with pcDNA3.1-CassN-Iag52B and pcDNA3.1-CassN-ICP2. Challenge was conducted 47 dpv. * indicates a statistical difference between the empty vector group and both the control group and the group injected with the combination subepidermally.

Results obtained from trial two (T2), in which rainbow trout were immunized with plasmids encoding membrane bound Iag52B and ICP2 and subsequently challenged with *I. multifiliis* showed a significant lower number of parasites on the fish in the group vaccinated with the empty vector and the group vaccinated with pcDNA3.1- CassN-ICP2 (Fig. 6, Table 5). A significantly elevated *I. multifiliis* antibody response 6 days following challenge was observed in the combination group (Fig. 6, Table 5) compared to the other groups (t-test, p<0.05) except for the group (Grp 6) injected subepidermally with a combination of the plasmids. All groups had an elevated *I. multifiliis* specific antibody response 53 dpv (6 dpch). The ELISA data for the group injected subepidermally with a combination of the plasmids showed a slight elevation of the specific antibody production compared to the control group, the empty vector group and the pcDNA3.1- CassN-Iag52B group.

**Table 5:** Results obtained in T1–3.

Trial No:	Group	Protection	ELISA	qPCR	Exp.
Average					IHC
weight,					
temperature					
**T1:**	Grp 1: Control	−	−	Nd	
7,6 g	Grp 2: pcDNA3.1	−	−	Nd	−
15–17°C	Grp 3: pcDNA3.1-CassN-Iag52A	−	−	+	−
	Grp 4: pcDNA3.1-CassN-ICP2	−	−	+	−
	Grp 5: pcDNA3.1-CassN-Iag52A	−	−	+	−
	+pcDNA3.1-CassN-ICP2				
**T2:**	Grp 1: Control	−	−	Nd	Nd
47 g	Grp 2: pcDNA3.1	−	−	Nd	−
15–17°C	Grp 3: pcDNA3.1-CassN-Iag52B	−	−	Nd	−
	Grp 4: pcDNA3.1-CassN-ICP2	−	−	Nd	−
	Grp 5: pcDNA3.1-CassN-Iag52B	−	+	Nd	−
	+pcDNA3.1-CassN-ICP2				
	Grp 6: pcDNA3.1-CassN-Iag52B	−	−	Nd	−
	+pcDNA3.1-CassN-ICP2				
**T3:**	Grp 1: pVax	−	−	Nd	−
5 g	Grp 2: pcDNA3-vhsG	−	−	Nd	+
11–14°C	Grp 3: pVax-CassL-Iag52B-EEF	−	−	Nd	+
	Grp 4: pcDNA3.1-CassN-ICP2	−	−	Nd	−
	Grp 5: pVax-CassL-Iag52B-EEF	−	−	Nd	+
	+pcDNA3.1-CassN-ICP2				

Different results obtained after three DNA vaccination trials in rainbow trout against *Ichthyophthirius multifiliis* with regard to protective effect (no significantly lower parasite burden in the target groups), *I. multifiliis* specific antibody production, significant mRNA regulations by qPCR and I-antigen protein detection by IHC staining with the primary antibodies; rabbit anti-G5II Iag52B and the monoclonal mouse *I. multifiliis* (I-ag) specific antibody MAb G3–61. All groups were injected im, except for Grp 6 in T2 which was injected subepidermally. Grp = group, Exp. = expression, + = stands both for a significantly elevated reaction and for a positive reaction, Nd = not determined.

**Figure 7:**
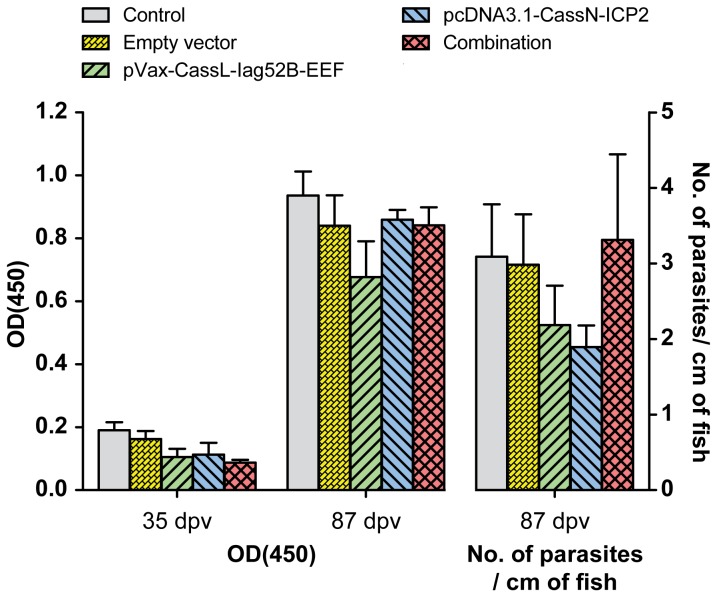
Antibody measurements and parasite load after challenge from T3. The graph shows both the measurement by OD(450) of *I. multifiliis* specific antibodies and the parasite burden of *O. mykiss* following challenge with *I. multifiliis* after DNA vaccination with pVax-CassL-Iag52B-EEF and pcDNA3.1-CassN-ICP2. Challenge was conducted 80 dpv. No statistical differences were observed between the groups.

Results obtained from trial three (T3), in which rainbow trout received an im injection of plasmids encoding a secreted form of Iag52B and a membrane bound form of ICP2: no protective effect was observed and no difference in specific antibody levels was observed between the groups (Fig. 7, Table 5).

In trial 4 (T4) im injections of Iag52A or B with or without the VHS DNA vaccine as a molecular adjuvant were tested. Mortality started 22 dpch, and 44 dpch all fish in the experiment had died. No difference in mortality between the groups was observed apart from a minor delay in the group injected with a mixture of pcDNA3.1-IAG52B and pcDNA3.0.

In trial 5 (T5) im injections of plasmids encoding Iag52B or Iag52B+A were compared with a needle free delivery method. The fish were exposed to the challenge infection in two replicate aquaria. Both of the aquaria contained a very high infection pressure and the mortalities commenced already at day three in aquarium two and at day 4 in aquarium one. Mortality was seen in all groups and all the fish had died at day 6 in aquarium two and at day 10 in aquarium one. A weak non-protective antibody production was found with western blotting when the vaccine included plasmids encoding both Iag52A and Iag52B.

In trial 6 (T6) im injections of Iag52A+B, membrane bound or secreted, with or without the complement fragment C3d as molecular adjuvant were compared with a gene gun delivery method of the same plasmids. The fish were exposed to the challenge in two separate aquaria. None of the plasmids used in T6 provided any protection against *I. multifiliis* following im injection and like the Panjet, no effect was seen after injecting the vaccines by gene gun. The mortality in aquarium one started at day 11 and after 21 days all the fish had died. In aquarium two the mortality began at day 15 and the experiment was terminated after the all fish had died at day 29.

No significant protective effect in any of the groups following vaccination was observed in trials 1–6.

### Immunohistochemical examination of the vaccination site following im injection

**Figure 8:**
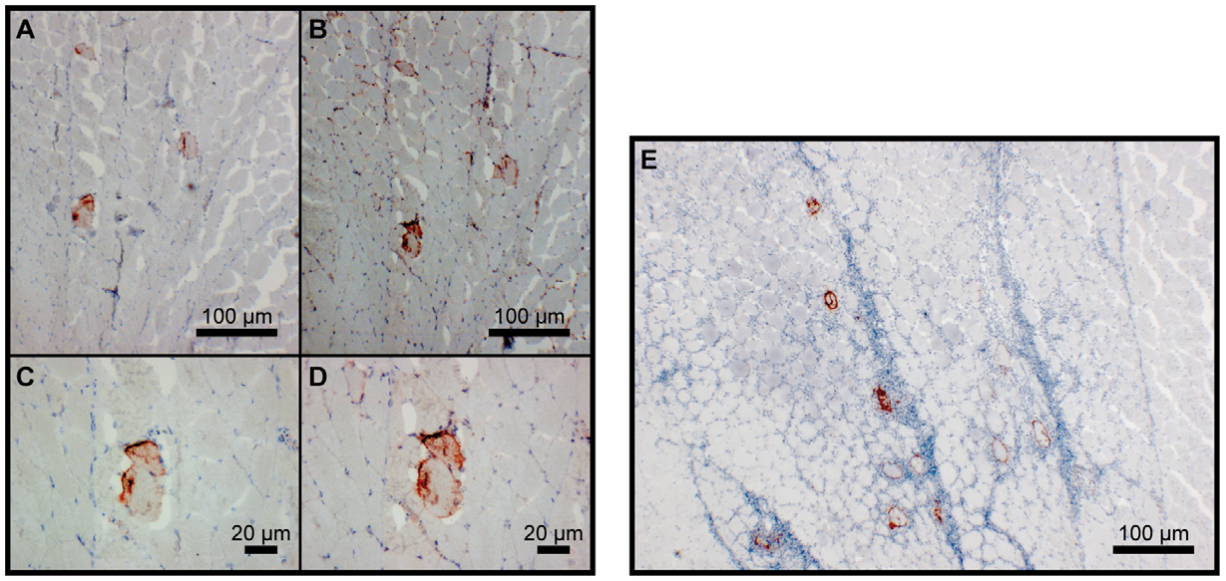
Immunohistochemical staining of muscle tissue from vaccinated fish. The images show the staining of *O. mykiss* muscle tissue at the injection site following intramuscular DNA vaccination with a mix of pVax-CassL-Iag52B-EEF and pcDNA3.1-CassN-ICP2 in T3; A–D) are images of tissues injected with pVax-CassL-Iag52B-EEF and pcDNA3.1- CassN-ICP2 while E) is an image of tissue injected with pcDNA3.0-vhsG. A, C and E) are from slides stained with MAb IP1H3 detecting the VHSV G carrier part, while B and D) are from slides stained with polyclonal rabbit anti-G5II Iag52B.

**Figure 9:**
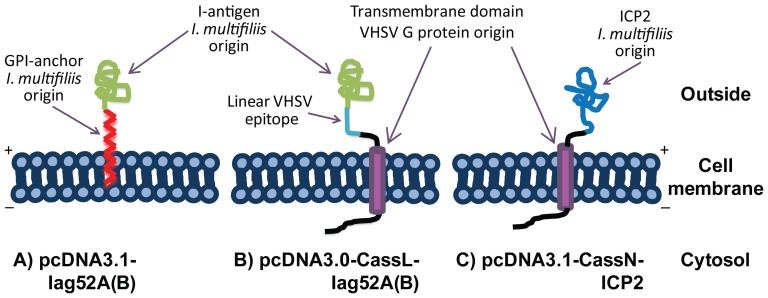
Interpretation of the *in silico* predicted transmembrane antigens encoded by selected plasmids. A) pcDNA3.1-Iag52A(B), B) pcDNA3.0-CassL-Iag52A(B) and C) pcDNA3.1-CassN-ICP2.

Immunohistochemical studies were performed on samples from T1–T3 only. The results confirmed the observations from immunostaining of transfected cell cultures. Among the tested constructs none of the CassN variants resulted in a positive antigen staining, while tissue from fish injected with pVax-CassL-Iag52B-EEF, encoding a secreted fusion protein with Iag52B flanked by VHSV G localization signals and MAb IP1H3 tag, stained positive with antibodies to both Iag52B and the MAb IP1H3 tag (Fig. 8A–E). Inflammation in tissues injected with plasmids encoding *I. multifiliis* proteins were never as pronounced as observed in tissues injected with plasmids encoding the surface G protein from VHSV (Fig. 8E) even though some inflammation was seen in the combination group. Successfully transfected cells in the muscle tissues were more numerous in the vhsG group compared to the other groups. A schematic interpretation of the *in silico* predicted transmembrane proteins, encoded by selected plasmids are shown in Figure 9.

## Discussion

The promising results obtained from DNA vaccination trials in rainbow trout with plasmid constructs encoding the fish rhabdovirus G protein [45] prompted us to try a similar vaccination strategy against white spot disease caused by the ciliate parasite *I. multifiliis*. Although rhabdoviruses and *I. multifiliis* are very different pathogens, some interesting parallels exist in terms of the surface glycoproteins. Monoclonal antibodies to conformational epitopes on the VHSV G protein and the *I. multifiliis* I-ags have both been reported to provide passive immunity following intraperitoneal administration of purified antibodies [21], [46]. Vaccination trials with recombinant and/or purified protein antigens have similarly identified the two proteins as inducers of neutralizing or immobilizing antibodies with assumed protective effect [23], [47], [48].

In the current study, a number of DNA vaccination strategies have been tested to determine the potential of genes encoding I-ags or a parasite derived cysteine protease as antigens in DNA vaccines against *I. multifiliis* in rainbow trout. Plasmid constructs with two I-ag variant (Iag52A and Iag52B) genes including the native gene sequences codon optimized for expression in vertebrate cells were described earlier [25]. These were tested alone or together and in combination with the vhsG. Combinations included both co-injection of the I-ag constructs with the reference VHS DNA vaccine encoding the full length VHSV G protein as molecular adjuvant as well as two different fusion gene constructs in which the central part of the I-ags was inserted between the terminals of vhsG. Since VHSV G is a transmembrane glycoprotein exposed on the host cell surface, the latter aimed at using the G protein terminals for cell surface display or secretion of the I-ags from transfected cells, and hereby stimulating the antibody response. Two different G protein display cassette constructs were tested, both including the G protein secretion signal (instead of the I-ag counterpart). The CassN constructs included a shorter part of the G protein C-terminal comprising the cytoplasmic and transmembrane domains only, while the CassL constructs further included the 100 membrane-proximal aminoacids of the extracellular part of the G-protein. The CassL version was further designed for secretion by omitting the transmembrane and cytoplasmic domains. The Cass N constructs was designed with a reduced vhsG part in an attempt to increase the immunogenicity of the I-ags and to direct the immune response solely to the I-ag parts.

### Antigenicity

Apart from the CassN fusion gene variants, all plasmid constructs were confirmed to mediate expression of the recombinant proteins in transfected fish cells for the Iag52B variant. Since mRNA transcripts could be detected at the injection site following vaccination with the CassN fusion gene constructs, the failure to detect the related proteins could either be due to lack of translation or insufficient quantity or quality of the recombinant proteins. Since both the VHSV G transmembrane anchor and the I-ag GPI anchor were included in these constructs, adverse membrane- or protein hydrophobic interactions may have interfered with protein expression, processing or stability.

Our results from immunostaining of cell cultures transfected with the original pcDNA3.1-Iag52A/B constructs prepared by Lin et al. (2002) [25] as well as the CassL constructs were partly in agreement with earlier observations on transfected mammalian COS-7 cells cultures by Lin and colleagues. The major difference concerned recognition of Iag52A and B protein variants by the immobilizing MAb G3–61. Lin et al. (2002) observed binding of the Iag52A variant only [25], while we in the present study could only detect the Iag52B variant. We addressed whether this could be explained by temperature related differences in protein expression levels or processing in the human COS-7 cells at 37°C and our fish EPC cells at 18°C, by immunostaining transfected human 293-T cells kept at 37°C. The staining profiles were as in EPC cells at 18°C (Fig. 2), suggesting other parameters than temperature to cause the differences. However, since MAb G3–61 recognizes a conformational epitope our results suggest that the EPC cells express a correctly folded Iag52B. Also, based on the staining of live transfected cells, where antibodies only have access to the extracellular space both the native GPI-anchored protein and the chimeric CassL fusion proteins were displayed in correct conformation on the surface of transfected fish cells. This suggest that posttranslational processing of the protozoan I-ag proteins worked correctly in the fish cells, although differences in glycosylation cannot be excluded.

Several interesting results were obtained in this study following immunization of rainbow trout with the different DNA vaccine combinations.

1. Immune-relevant gene regulations were observed following injection of plasmids (T1). Most importantly, when a divalent vaccine was used (plasmids encoding Iag52A and ICP2 respectively) the most extensive gene regulations were seen.
2. A weak *I. multifiliis*-specific antibody response was induced following injection of a mix of genes encoding an I-ag and a cysteine protease (T2).
3. It was possible to obtain an *in vivo* positive transfection of rainbow trout muscle cells (T3). The cells, which were transfected, did not induce an inflammatory response.
4. In spite of different injection techniques, with or without C3d or the VHS DNA vaccine as “molecular” adjuvants or carrier, no protective effect against *I. multifiliis* was obtained (T4–6).

### 1) Gene regulations (Trial T1)

Despite failure to detect expressed antigen in transfected cells, several responses to vaccination were observed at gene expression level. An up-regulation of the B-cell receptor gene IgM (secreted) was observed, which indicates increased B-cell activity. A sudden drop in the expression of IgM is observed 1 dpch for the combination group and a following increase is seen for the three target groups (3–5) 7 dpch. The drop of IgM expression could be due to the fish immune system focusing on acute mucosal immunity where the infection takes place or that the fish becomes stressed. The rise of the expression 7 dpch seen only for the three vaccination groups may be due to recognition of the proteins on the parasite used for vaccination. These results could be interpreted as infiltration of lymphocytes at the injection site suggesting both a cellular and a humoral immune response corresponding to observations in other DNA vaccination trials [45], [49]. However, this would be contradicting to what we saw in the IHC analyses. The expression of two markers (MHC I and II) associated with antigen presentation were slightly up-regulated at the injection site on day 14 and also on day 32 for MHC II in all groups. All self and foreign peptides will be presented by these two molecules and up-regulations of the expression of these genes are known to take place at injection sites in fish vaccinated with rhabdovirus G-gene DNA vaccines [50], [51]. An up-regulation of MHC II could indicate infiltration of professional antigen presenting cells. Vector DNA by itself is immunostimulatory [50] due to unmethylated CpG dinucleotides and can serve as an adjuvant and induce responses. However, in the qPCR analyses presented here little or no response towards the vector alone was observed. The increase of TCR β mRNA at the injection site 14 and 32 dpv could indicate that T cells accumulate or increase expression in the area, especially when a mixture of plasmids encoding Iag52A and ICP2 was injected. However, neither protection nor a detection of *I. multifiliis* specific antibodies was observed. A possible explanation could be that the proteins encoded by the inserted DNA sequences (Iag52A and ICP2) were expressed in insufficient amounts or quality since the encoded *I. multifiliis* proteins could not be detected in transfected cells. Lin et al. (2002) also found that their version of full length Iag52A gene inserted into pcDNA3.1 did not produce *I. multifiliis* specific antibodies in channel catfish. Only a secreted version of the Iag52A gene insert resulted in the production of antibodies [25].

Results from the expression studies of the spleen show regulations of IFN-γ and IL-10 at day one and day 56 with the most regulation in the group given a mixture of plasmids encoding Iag52A and ICP2. IFN-γ has antiviral, immunoregulatory and anti-tumor properties and is associated with Th1 responses [52], [53] while IL-10 is an anti-inflammatory cytokine associated with Th2 responses [54]. Regulation of these two molecules indicates an activation and regulation of the immune system. In the liver the genes encoding SAA and hepcidin were up-regulated following challenge, with the group given the mixture of plasmids encoding Iag52A and ICP2 reaching the highest fold change. A previous study showed up-regulations of these two genes following injection of live *I. multifiliis* theronts in the peritoneal cavity, which led to protection [6]. However, the DNA injections in this trial did not induce regulation and it is possible that these two acute phase proteins are essential for the induction of protective immunity against *I. multifiliis* [6], [55]. The expression analyses indicate that the highest regulations are seen in the group given the mixture of plasmids encoding Iag52A and ICP2. The immune system appears to react stronger when two different genes encoding different proteins are introduced even though the injected amount of DNA was the same as in the other groups. This may indicate that it is more immune stimulating to do immunizations with a combination of several proteins as recognized by other authors [56], [57].

### 2) Antibody responses

Already within 10 days after an *I. multifiliis* infection fish start to produce specific antibodies against the parasite [58], [59]. Accordingly, 6 days post challenge in T2 all groups had an elevated *I. multifiliis* antibody production. However, between subgroups, significantly elevated antibody reactivity was observed for the combination group with fish injected with a mixture of plasmids encoding membrane bound Iag52B and ICP2. This increase far from reached that of an immune rainbow trout (with OD(450) readings ranging from 2–4), which might explain the lack of protection in the vaccinated fish. Even though no positively transfected cells were observed the antibody reactivity suggests that some antigen expression has taken place.

### 3) In vivo positive transfections

Expression of a secreted form of the *I. multifiliis* surface protein Iag52B was confirmed both in the transfection studies of EPC cells and in the IHC analyses of *in vivo* transfected rainbow trout muscle cells (T3). It was observed from the IHC staining that positively transfected cells did not induce a heavy inflammation as seen for vhsG transfected cells (Fig. 8A, 8B and 8E). This lack of inflammation might be due to secretion rather than cell surface exposure of the antigen, since a secreted version of VHSV G also induced little inflammation (unpublished observations). Whether the lack of induced inflammation can explain the poor immune response remains to be determined. However, some kind of adjuvant effect is likely beneficial for the immune response to *I. multifiliis* antigens, based on earlier observations where immunization with the *I. multifiliis* G5 purified I-ag together with Freund's complete adjuvant induces protection whereas immunizations with the I-ags alone do not [23].

### 4) Delivery and immunogenicity

Plasmids encoding membrane bound Iag52A and Iag52B were co-injected with the VHS DNA vaccine as a molecular “adjuvant” (T4). This was based on earlier experiments indicating that expression of the VHSV G protein induces a local inflammation, which potentially could mediate an efficient activation of the immune system [12]. However, no protection was obtained following im vaccination with the combined plasmids. Plasmids encoding Iag52A and Iag52B coupled to a membrane region of VHSV G were injected im or by the use of a needle free injector (T5). The plasmid encoding Iag52B was either injected alone or in combination with Iag52A. The Panjet was used as an attempt to inject the vaccines at the site of infection (i.e. the skin), and has previously been shown to be able to induce short term protection against VHS following vaccination with the VHS DNA vaccine (unpublished observations). However, none of the vaccine constructs or delivery methods induced significant protection against *I. multifiliis* although a small delay in the development of mortality was observed along with a weak antibody response when plasmids encoding both I-ags were used. However, the infection pressure in this trial during challenge might have been too high resulting in the fish dying from an acute reaction on the initial infection rather than from the classical disease. The gene gun has been used with great success in both mammals and fish to deliver plasmid DNA intradermally and thereby obtain protective immunity against viral and parasitic pathogens [60], [61]. Plasmids containing secreted variants of the I-ags and plasmids containing secreted variants of the I-ags combined with three copies of the C3d fragment of the complement protein C3 were included in one trial (T6). The addition of a gene sequence encoding 3xC3d in the 3′ end of a secreted vaccine gene has previously been shown to mediate 10 to 1000 fold improved efficacy of a DNA vaccine, possibly due to an opsonizing effect of C3d mediating uptake in antigen presenting cells [28].

None of the DNA vaccination strategies tested in this study provided significant protection against *I. multifiliis*. Since proper antigen expression was demonstrated for several plasmid constructs, a lack of immunogenicity of the antigens when delivered as DNA vaccines and hereby a lack of *I. multifiliis* specific antibodies may explain the absence of protection. Earlier analyses have demonstrated a rather high variability in the expression levels of the different I-ags by the individual *I. multifiliis* parasites [62] and although the parasites used for ELISA and for challenge in the present study appeared to belong to the same serotype as the ones providing the antigen gene sequences [25], it may not be excluded that our failure to induce protection could also be related to quantitative or qualitative differences in vaccine and challenge I-ags.

### Conclusions

The lack of protective effect in the current DNA vaccination trials against *I. multifiliis* contrasts the high protection obtained with the rhabdovirus DNA vaccines and it may reflect a basic difference between the immune reactions required to protect against the systemic viral infections compared to the skin infection of *I. multifiliis*. The early IFN-related protection induced by the viral G gene vaccines have been demonstrated to protect across viral pathogens, but not against the bacterial pathogen *Yersinia ruckeri* [63]. Accordingly, initial experiments in the present study also confirmed that the early protection did not cover *I. multifiliis* (unpublished observations). While rhabdoviruses like VHSV cause a systemic infection and pathological effects throughout the organs and tissues of the fish, the *I. multifiliis* infection mainly affects the skin and the gills. This implies that while a systemic immune response is required for protection against VHS, both systemic and mucosal immune responses may be required for protection against *I. multifiliis* [33], [64]. Accordingly, passive immunization experiments in which serum from immunized donor fish were ip injected into recipient fish before challenge conferred protection against VHS but not against *I. multifiliis* [19], [34], [51]. The latter observation seems contradictory to the protective effect of ip injected immobilizing MAbs, but was explained by a potentially higher tissue penetration power of the monomeric mouse IgG compared to pentameric mouse IgM and tetrameric fish IgM [34]. Although the relationship between serum and mucus Ig responses in fish remain to be understood in detail [65], [66], it has been shown that immunization by ip injection can induce both systemic and mucosal antibodies [67]. Accordingly, mucosal protection against *I. multifiliis* can arise from systemic immunizations [6]. However, the work by Swan et al. (2008) suggested that mucus IgM was derived from skin associated antibody producing cells. Whether DNA vaccines as applied here are able to induce a mucosal antibody response in fish remains to be determined, but their failure to induce any protection against *I. multifiliis* in the current study might suggest this to be the case. A recently discovered immunoglobulin class in teleost fish known as IgT is believed to be particularly important in mucosal immunity and *I. multifiliis* binding IgT antibodies have also been demonstrated in the mucosa of immune fish [33], [66]. Mucosal immunization might be required to reach a high IgT response [68]. In the present study, sub-epidermal and epidermal delivery of DNA vaccines was attempted by jet spray, gene gun and injection just beneath the epidermis, but it may be anticipated that jet spray and gene gun techniques penetrate rather than stimulates the mucosal barrier. Apart from further search for vaccine candidate antigens, future attempts to generate protective immunity in fish by vaccination against *I. multifiliis* should focus on resolving and stimulating protective mucosal immune mechanisms either by optimized systemic immunization or by a targeted mucosal delivery.
